# The Neuromuscular Fatigue-Induced Loss of Muscle Force Control

**DOI:** 10.3390/sports10110184

**Published:** 2022-11-21

**Authors:** Jamie Pethick, Jamie Tallent

**Affiliations:** 1 School of Sport, Rehabilitation and Exercise Sciences, University of Essex, Colchester CO4 3SQ, UK; 2 Department of Physiotherapy, School of Primary and Allied Health Care, Faculty of Medicine, Nursing and Health Science, Monash University, Melbourne 3800, Australia

**Keywords:** neuromuscular, fatigue, motor control, force control, steadiness, entropy, fractal, motor unit, synaptic input

## Abstract

Neuromuscular fatigue is characterised not only by a reduction in the capacity to generate maximal muscle force, but also in the ability to control submaximal muscle forces, i.e., to generate task-relevant and precise levels of force. This decreased ability to control force is quantified according to a greater magnitude and lower complexity (temporal structure) of force fluctuations, which are indicative of decreased force steadiness and adaptability, respectively. The “loss of force control” is affected by the type of muscle contraction used in the fatiguing exercise, potentially differing between typical laboratory tests of fatigue (e.g., isometric contractions) and the contractions typical of everyday and sporting movements (e.g., dynamic concentric and eccentric contractions), and can be attenuated through the use of ergogenic aids. The loss of force control appears to relate to a fatigue-induced increase in common synaptic input to muscle, though the extent to which various mechanisms (afferent feedback, neuromodulatory pathways, cortical/reticulospinal pathways) contribute to this remains to be determined. Importantly, this fatigue-induced loss of force control could have important implications for task performance, as force control is correlated with performance in a range of tasks that are associated with activities of daily living, occupational duties, and sporting performance.

## 1. Introduction

It has been recognised by perceptive observers since antiquity, that intensively exercised muscles show a progressive decline in performance, a phenomenon traditionally termed neuromuscular fatigue [1] and more recently termed performance fatigability [2]. Neuromuscular fatigue can be broadly defined as an exercise-induced decline in maximal force (or torque)-generating capacity [3]. Performance fatigability extends this definition to be a decline in any objective measure of performance over a discrete period of time [2], thus, recognising that the capacity to *generate* maximal muscle force is not the sole determinant of exercise performance. Indeed, the ability to *control* submaximal muscular forces, i.e., to generate task-relevant and precise levels of force [4], is also an important, though often overlooked, factor in determining performance [5].

It has long been known that variability is an unavoidable feature of voluntary muscle contraction [5,6,7]. Consequently, muscle force output is neither smooth nor steady; rather, it exhibits constant inherent fluctuations around the required target force [4,8], indicating that control of force is not perfectly accurate [9]. An effect of neuromuscular fatigue on the ability to control force was first commented on in 1920 by Leon Binet, who stated that “tremor increases as a result of muscular contraction and becomes exaggerated under the influence of work” [10]. A century of subsequent research has demonstrated increases in the magnitude [11,12,13] and, more recently, decreases in the temporal structure (i.e., complexity) [14,15,16] of muscle force fluctuations during fatiguing contractions, with both of these changes being indicative of a poorer ability to control force [4,8].

Recent research has focused on the mechanistic basis of the neuromuscular fatigue-induced decrement in submaximal muscle force control, suggesting that, as with the mechanisms underpinning the decrement in maximal force-generating capacity, both central and peripheral processes may be involved [17,18]. However, of equal, if not greater, importance is the effect the loss of muscle force control has on exercise performance, where it serves to hinder the ability to exert a desired force and to produce an intended movement trajectory [19]; this explains a significant amount of variance in the performance of functional tasks (e.g., static [20] and dynamic balance [21]), and has been postulated to be of relevance for exercise tolerance [22]. The fatigue-induced loss of muscle force control could, therefore, restrict performance in a range of populations and tasks, from occupational duties (e.g., firefighters, surgeons), to older adults performing activities of daily living, to professional athletes.

The purpose of this review is to provide a detailed overview of neuromuscular fatigue-induced changes in muscle force control. This examination first necessitates a brief description of the measurement and quantification of muscle force control during neuromuscular fatigue. We then provide evidence regarding how muscle force control is altered with neuromuscular fatigue, and outline the potential mechanistic basis. We finish by discussing the performance implications of a neuromuscular fatigue-induced loss of muscle force control. Throughout the review, we also highlight limitations of previous research and gaps in our knowledge (and, therefore, areas for future research to focus on) regarding the neuromuscular fatigue-induced loss of muscle force control.

## 2. Measurement and Quantification of Force Control during Neuromuscular Fatigue

**Figure 1:**
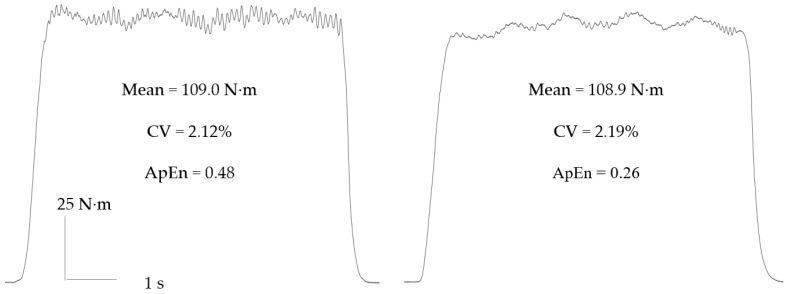
Constant inherent fluctuations during isometric contractions performed at 40% maximal voluntary contraction (MVC). The two contractions have near identical means and magnitudes of variance (i.e., coefficient of variation, CV), but distinctly different dynamics. They can only be discriminated with the use of complexity (i.e., approximate entropy, ApEn) measures.

Muscle force control is typically measured using isometric contractions at an imposed target, reflecting a percentage of participants’ maximal voluntary contraction (MVC) [4,23]. Such isometric contractions allow the fixation of the limb/muscle group in question, and serve to eliminate any extraneous sources of fluctuation [12]. During these isometric contractions, the exerted force will fluctuate around the target (Figure 1). These fluctuations can then be quantified according to their magnitude and/or complexity. Detailed descriptions of these magnitude- and complexity-based measures can be found elsewhere [23,24,25,26].

In brief, traditional magnitude-based measures provide an index of the degree of deviation from a fixed point within a time series [8]. The standard deviation (SD) quantifies the absolute magnitude of fluctuations in muscle force output, while the coefficient of variation (CV) quantifies the magnitude of fluctuations normalised to the mean force output, thus facilitating comparisons between individuals/populations that differ in maximal strength [23]. These measures reflect force steadiness [4]. It must be noted, though, that force control, and the magnitude of force fluctuations, have only been calculated in this way since around the turn of the millennium [27,28]. Prior to that, force control was often quantified according to the frequency [12,29] or root mean square [30,31] of physiological and/or force tremor.

A limitation of magnitude-based measures is their failure to discriminate outputs with distinctly different dynamics (Figure 1) [24]. Complexity-based measures characterise the moment-to-moment relationship between successive points (or series of points) in an output [32], thereby allowing the quantification of properties such as temporal irregularity, time irreversibility and long-range fractal correlations [33]. Approximate entropy (ApEn) and sample entropy (SampEn) quantify the degree of regularity/randomness in an output [32,34], while detrended fluctuation analysis (DFA α) provides a measure of long-range fractal correlations within an output [35]. These measures reflect force adaptability, i.e., the ability to adapt force output rapidly and accurately in response to perturbations [18]. Due to the differing information provided by magnitude- and complexity-based measures, it has been recommended that the two approaches be used in conjunction to provide a more complete understanding of force control [4,23].

**Figure 2:**
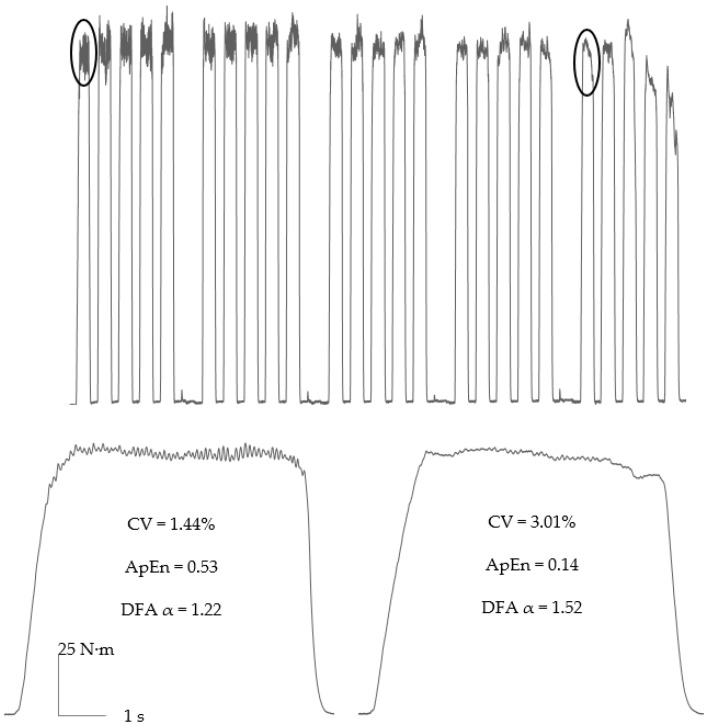
An intermittent isometric fatigue test (6 s contraction, 4 s rest duty cycle) performed in the knee extensors at 40% of MVC. Contractions circled in the top panel are expanded below. There is a progressive increase in the magnitude (i.e., CV) and decrease in the complexity (i.e., ApEn; detrended fluctuation analysis, DFA) of knee extensor force fluctuations.

The isometric contractions used to measure force control also represent a useful model with which to elicit neuromuscular fatigue and study its mechanisms, as techniques including electromyography and electrical stimulation can easily be applied [14,36]. The majority of current studies have measured muscle force control during a variety of sustained and intermittent (e.g., Figure 2) isometric protocols, using both maximal and submaximal contractions, and typically performed to task failure [14,18,37]. Such protocols have the advantage of continuously measuring muscle force control, thus allowing the development of neuromuscular fatigue-induced changes in force control to be tracked. However, given that isometric fatigue tests are contrived forms of exercise that are unique to laboratory settings, it could be argued that these protocols lack ecological validity, and that there is a need, instead, for studies to investigate force control prior to and after performing the type of dynamic (i.e., concentric and eccentric) contractions typical of sporting and everyday actions. Indeed, studies that utilised such protocols have demonstrated differences in force control, as a result of different types of fatiguing muscle action [38,39,40].

## 3. Neuromuscular Fatigue-Induced Changes in Force Control

Over 100 years have passed since Binet’s initial (1920) assertion regarding the effects of muscular contraction and the influence of work on tremor [10]. The first experimental test of this assertion was conducted by Bousfield in 1932 [11]. Utilising a kymograph to measure index finger tremor and a repeated wrist flexion protocol to induce neuromuscular fatigue, it was observed that “the rate, amplitude, and irregularity of tremor oscillations vary directly with the degree of fatigue”. This statement is of particular interest, as the amplitude (or magnitude) of tremor and force fluctuations has been frequently studied in the interim [12,13,29,30], but it was not until much more recently (2015) that changes in the irregularity (i.e., complexity) of force fluctuations with neuromuscular fatigue were quantified [14]. Nevertheless, since Bousfield’s observations, numerous studies have demonstrated changes in the magnitude and complexity of force fluctuations during fatiguing contractions, with these changes being dependent on multiple factors, including contraction type, contraction intensity, the sex and age of participants and the administration of ergogenic aids.

### 3.1. Contraction Type

Initial studies used sustained isometric contraction protocols, with a given submaximal intensity contraction that was held for either a fixed time or until task failure (i.e., the point at which participants deviated from the target by a set amount for a specific period of time), in order to both induce neuromuscular fatigue and measure its effect on force control [12,27,28,29,30,31,37,41]. Such studies have observed progressive increases in the magnitude of force fluctuations, as quantified by measures such as the root mean square of force tremor [12,29,30,31] and the CV of force [27,28,37,41]. Moreover, these increases in the magnitude of force fluctuations have been observed in a variety of muscle groups, ranging from small upper limb muscles associated with dextrous movement (e.g., index finger abductors) [12,29], to large lower limb muscles associated with locomotion (e.g., knee extensors) [27,37]. This frequently observed increase in the magnitude of force fluctuations serves to increase targeting error, and is reflective of decreased (i.e., poorer) steadiness. A progressive decrease in the complexity of force fluctuations, as quantified by ApEn, SampEn, and DFA α, has also been observed during isometric contractions sustained to task failure in the knee extensors [37] and elbow flexors [42]. This loss of complexity is manifest as fluctuations in force becoming more regular/predictable, and is reflective of decreased adaptability to external perturbation (such as changing force demands during typical motor activity). Both an increase in the magnitude and a decrease in the complexity of muscle force fluctuations are indicative of a loss of force control, and have important functional consequences, given that the accuracy of voluntary movements is often more important for successful performance than maximal force-generating capacity [5,20,21].

The studies mentioned above can, however, be criticised somewhat for their use of sustained contraction protocols to induce neuromuscular fatigue. It could be argued that sustained contractions are not reflective of the type of muscle contractions that are performed in daily life and sporting events, which tend to be characterised by intermittent (e.g., the contraction/relaxation cycles of walking, running) rather than continuous force production [43]. Furthermore, sustained contractions increase mechanical occlusion of the vasculature supplying the muscle and, consequently, decrease muscle perfusion [44]. Indeed, muscle reperfusion has been observed to stop at forces as low as 10–25% of MVC during sustained contractions [45,46]. Thus, sustained contractions have the potential to exacerbate peripheral fatigue via metabolite accumulation (inorganic phosphate, ADP, H^+^ and/or K^+^), such that its effects on force control are out of proportion to that experienced during the more intermittent movement patterns of daily life and sporting performance.

Intermittent isometric contraction protocols, in which holding a given intensity contraction is interspersed with regular rest periods (to allow muscle reperfusion) and performed until task failure [14,15,44], may be more representative of typical patterns of muscle activation and neuromuscular fatigue development [43]. Protocols using such intermittent fatiguing contractions (typically with a duty cycle of 60%; e.g., 6 s contraction, 4 s rest or similar) have also demonstrated an increase in the magnitude [14,47,48] and a decrease in the complexity [14,22,47] of force fluctuations in the knee extensors (Figure 2), although, as discussed further below, this effect is dependent on contraction intensity [15,18,48].

Interestingly, several studies have compared sustained and intermittent isometric contractions at the same contraction intensity, finding evidence to support the suggestion that sustained contractions may overestimate the effect of neuromuscular fatigue on muscle force control. Madeleine et al. [49] observed no change in elbow flexor force CV at 30% of MVC during 30 min of intermittent contractions (6 s contraction, 4 s rest), whereas a progressive increase in CV was observed during a sustained contraction, for which task failure occurred in <5 min. Similarly, Kavanagh et al. [44] observed no change in elbow flexor force CV at 20% of MVC during 600 s of intermittent contractions (5 s contraction, 5 s rest), whereas a progressive increase in CV was observed during a 600 s sustained contraction. Importantly, the intermittent contractions were accompanied by no change in elbow flexor-evoked resting twitch force (a measure of peripheral fatigue), while the sustained contraction was accompanied by a progressive decrease in resting twitch force, indicating the development of peripheral fatigue.

Further studies have investigated muscle force control in response to neuromuscular fatigue protocols, utilising dynamic concentric or eccentric contractions. Given the preponderance of such muscle actions in activities of daily living and sporting events, these contractions may represent a more ecologically valid modality to induce fatigue than using intermittent (or sustained) isometric contractions. Semmler et al. [50] compared isometric force control after fatiguing concentric and eccentric elbow flexor exercise. No change in muscle force CV was observed at any contraction intensity (5, 25, 35 and 50% of MVC) after the concentric exercise, whereas significant increases in CV were observed at all contraction intensities after eccentric exercise. This study was, however, subject to several limitations. Firstly, there was a disparate decrement in MVC force following the concentric (−22% of MVC) and eccentric (−45% of MVC) exercises; secondly, eccentric exercise, particularly when unaccustomed to, leads to both neuromuscular fatigue and muscle damage, the latter of which may influence force control independently of fatigue. To combat both of these limitations, Ye et al. [38,39] used resistance-trained participants, who were more accustomed to muscle-damaging exercise, and volume-matched concentric and eccentric exercise protocols that resulted in similar MVC losses. Isometric force CV during contractions at 40% of MVC increased following both concentric and eccentric exercise, though this increase was significantly greater after the eccentric exercise. Muscle force complexity, as quantified by ApEn and DFA α, has also been demonstrated to decrease following eccentric exercise, with this deficit lasting longer than 60 min after exercise cessation [51]. In comparison, complexity had recovered by 10 min after the cessation of isometric exercise that induced the same decrement in MVC.

The above studies demonstrate that the type of dynamic contractions typical of daily life and sporting events can result in a loss of force control. It must be noted, though, that the concentric and eccentric contractions were still somewhat contrived, having been performed on an isokinetic dynamometer at a controlled angular velocity [38,39,51]. Currently, there is a paucity of studies that involve fatiguing exercises performed outside of an isokinetic dynamometer. Singh et al. [52] investigated the effect of (a minimum of 88) barbell squats with an additional load of 40% body-weight on isometric knee extensor force steadiness, finding a significant increase in the magnitude of fluctuations, and a significant decrease in accuracy. Oliveira et al. [53] used a unilateral calf raise protocol (5 sets of 20 repetitions), finding a significant decrease in plantarflexor SampEn that remained depressed below baseline values for up to 60 min after exercise cessation. Further research is, however, needed on whether fatiguing locomotor activities (e.g., walking, running, cycling, etc.) affect muscle force control. This is important, given that locomotor activities (particularly walking) constitute a crucial component of daily living activities, and that many sports (not just track events) involve periods of high-intensity fatiguing running (e.g., team sports, such as football, rugby and hockey).

### 3.2. Contraction Intensity

The intensity of muscular contractions plays an important role in the development of neuromuscular fatigue and, therefore, has a significant effect on the consequent loss of force control. The critical torque (CT; analogous to the critical power measured during whole-body tasks) represents a critical neuromuscular fatigue threshold (or, as has recently been demonstrated, phase transition [54]) that separates the heavy and severe exercise domains [55,56]. During contractions performed above the CT, it is not possible to achieve a metabolic steady state, in contrast to contractions performed below it [56]. A consequence of this metabolic non-steady state is the development of peripheral fatigue ~4–5 times faster above CT compared with below it [18,54,55]. Indeed, metabolite-mediated peripheral is thought to be the dominant mechanism of MVC force losses above CT [56]. Thus, contraction intensity, specifically whether contractions are performed below or above the CT, can provide important insights into the mechanisms underpinning neuromuscular fatigue-induced loss of force control.

Studies that investigated muscle force control during sustained fatiguing contractions utilised contractions intensities of ≥20% of MVC, demonstrating an increase in the magnitude [13,30,31,41,44,47] and decrease in the complexity [37,42] of force fluctuations. Castronovo et al. [17] observed an increase in CV during sustained ankle dorsiflexion contractions at 20 and 50% of MVC, but not at 75%. Moreover, the strength of common synaptic input to motor neurons (thought to be the main determinant of muscle force fluctuations [9]) also significantly increased throughout the contractions at 20 and 50% of MVC. A similar load-dependence of neuromuscular fatigue was observed by Ebenbichler et al. [27], with increases in SD during sustained knee extension contractions evident at 30 and 50% of MVC, but not 70% of MVC. Further studies have, nevertheless, demonstrated increases in the SD and CV, and a decrease in ApEn, during contractions ranging from 80–100% of MVC [37,57]. During sustained contractions, the critical torque typically occurs at ~15% of MVC [58,59], suggesting that the contractions in the aforementioned studies were likely performed above the CT. To our knowledge, though, there are no studies on muscle force control involving sustained contractions that provide a comparison of intensities below and above the CT.

Several studies have, however, compared intermittent contractions below and above the CT [15,18,54], concluding that the CT is also a critical threshold for the neuromuscular-fatigue induced loss of muscle force control. Pethick et al. [15] measured knee extensor CT during intermittent contractions, finding it to be ~30% of MVC. During subsequent contractions performed at 50 and 90% of the calculated CT (i.e., at ~15 and 27% of MVC), there was no change in either the magnitude (SD, CV) or complexity (ApEn, SampEn, DFA α) of force fluctuations during 30 min of contractions. In contrast, contractions performed above the CT resulted in progressive increases in the magnitude and decreases in the complexity of force fluctuations, until task failure was reached (see Figure 2 in [18]). Moreover, at task failure, the magnitude of fluctuations had increased to similarly high values, and the complexity of fluctuations decreased to similarly low values, irrespective of the absolute demands of the task (i.e., contraction intensities between 37 and 58% of MVC) [15,54]. The loss of muscle force control only during contractions performed above the CT suggests that fatigue mechanisms particular to such contractions, i.e., metabolite-mediated peripheral fatigue, are involved. It has, in fact, been postulated that metabolite-mediated peripheral fatigue is a prerequisite for central adjustments that act on the motor unit pool, which are then responsible for changes in the strength of common synaptic input [22]. It must be noted, though, that a causal relationship between peripheral fatigue and the loss of force control has yet to be established.

### 3.3. Sex Differences

The observation of sex differences in force control during contractions in fresh muscle is equivocal, and may depend on the muscle group tested [44,48,60,61]. Both males and females do, however, exhibit an increase in the magnitude of force fluctuations during fatiguing contractions [13,44,48,57]. Due to the fact that females are typically less fatigable (i.e., take longer to reach task failure) than males during isometric contractions at the same relative intensity [62], the rate of increase in CV is significantly slower in females [48,57]. This slower rate of increase in CV in females is typically accompanied by a slower rate of decrease in MVC and a slower rate of increase in EMG activity [44,48], which are indicative of a slower rise in recruitment of additional larger motor units. The differences in the rates of increase in CV and EMG likely reflect sex differences in muscle metabolism and contractile properties [62] that contribute to the slower development of peripheral fatigue in females [44] and, consequently, less need for additional motor unit recruitment and/or increases in motor unit firing rates to maintain force output. Several studies have also recently demonstrated that the fatigue-induced increase in CV is greater in males than females [44,48].

Little research has investigated sex differences in the complexity of force fluctuations with neuromuscular fatigue. During intermittent knee extension contractions at 30% of MVC in older adults (aged ≥65 years), Mehta and Rhee observed an increase in SampEn in males only [63]. This increase in SampEn contrasts with the neuromuscular fatigue-induced decrease in complexity (as measured by ApEn, SampEn and DFA α) that was previously reported in young adults [14,18,22,42]. Such divergent findings could simply be reflective of differences in force control behaviour associated with ageing, although they are more likely to reflect differences in force sampling frequency and SampEn processing choices (“*m*”, the template length and “*r*”, the tolerance for accepting matches), which have the potential to effect both the pattern and magnitude of change in SampEn values [64]. It has been recommended that the optimal sampling frequency when investigating the complexity of isometric force control is >200 Hz, and that *m* is set to 2 and *r* to 0.1SD [64], a configuration used by much of the research on the fatigue-induced decrease in muscle force complexity [14,15,18,42]. In contrast, Mehta and Rhee [63] used a sampling frequency of 100 Hz, and set *m* to 4 and *r* to 0.2SD.

An additional consideration for female participants is whether hormonal oscillations due to menstrual cycles affect the neuromuscular fatigue-induced loss of force control. Currently, the limited research on this suggests that the mid-luteal phase of the menstrual cycle is associated with a greater magnitude of force fluctuations in unfatigued muscle, and that this may persist with the development of fatigue [65]. This was speculated to reflect a mediating action of progesterone, which has been demonstrated to decrease corticospinal excitability [66]. Although a small body of research is developing in regards to sex differences in neuromuscular performance, there is still only a superficial understanding of the differences in, and the mechanisms contributing to, sex differences in the neuromuscular fatigue-induced loss of force control.

### 3.4. Age Differences

It is well-established that older adults (aged ~60–75 years old) are less fatigable than young adults during isometric contractions performed at the same relative intensity [67]. This is accompanied by slower rates of increase in CV and EMG activity [68,69]. It has also been demonstrated that the magnitude of force fluctuations at the end of a fatiguing task is significantly greater in young compared to old adults [68,69], with this speculated to relate to an increase in EMG burst activity. Hunter et al. [68] observed a significant correlation between increases in elbow flexor force CV and EMG burst activity throughout the course of a fatiguing contraction, suggesting the increase in CV was explained by transient recruitment of additional motor units that were activated for brief bursts, in order to compensate for a decline in the force capacity of active muscle fibres.

Several studies have also demonstrated that baseline values for force control measures are predictive of endurance time in older adults. Justice et al. [70] found that variance in time to task failure for older adults during a submaximal isometric task is significantly predicted by age, force CV and strength [70], while Duan et al. [71] observed that adding the baseline CV and SampEn of knee extensor force to gender and obesity increased the explanatory power of a regression model for time to task failure in older adults from 16.2% to 49% [71].

### 3.5. Ergogenic Aids

Several ergogenic aids have been demonstrated to attenuate the neuromuscular fatigue-induced loss of force control, which could have important implications for the maintenance of task performance. Acute caffeine ingestion has been demonstrated to slow the neuromuscular fatigue-induced increase in the magnitude and decrease in the complexity of knee extensor force fluctuations during intermittent isometric contractions at 50% of MVC, consequent to a slower rate of decrease in force-generating capacity and slowed development of central fatigue [47]. Indeed, at “isotime”, the time point in the caffeine condition equivalent to task failure in the placebo condition, SD and CV remained significantly lower, while ApEn remained significantly greater, suggesting that force steadiness and adaptability were maintained for longer with caffeine ingestion. Similarly, seven days of New Zealand blackcurrant extract supplementation has been demonstrated to decrease knee extensor force CV, in comparison to a placebo, during the latter half of a sustained 120 s contraction at 30% of MVC [72]. Ischaemic pre-conditioning (an intervention consisting of alternating bouts of muscle ischaemia and reperfusion prior to exercise) has also been demonstrated to slow the neuromuscular fatigue-induced decrease in muscle force complexity (ApEn, DFA α), but not the increase in the magnitude of fluctuations (SD, CV) [73]. Further research into the effects of ergogenic aids on the fatigue-induced loss of force control is warranted. Any intervention that can slow the fatigue-induced loss of force control could help maintain performance of skilled movements for longer, and decrease risk of injury (discussed further below in Section 5. Performance implications of the neuromuscular fatigue-induced loss of muscle force control).

## 4. Mechanistic Basis of the Neuromuscular Fatigue-Induced Loss of Muscle Force Control

Motor units are the basic functional unit of the neuromuscular system, responsible for transducing synaptic input into voluntary force and movement [25]. Motor neurons receive both common and independent synaptic input, though the independent input (“synaptic noise”) is effectively filtered out while the common input is transmitted, approximately unaltered, to the output of the motor neurons [74]. Common synaptic input has, therefore, been postulated to represent the effective neural drive to muscle, and to be the main determinant of force fluctuations [74,75]. This is supported by the observation that the cumulative motor unit spike train, which represents the common synaptic input to the motor neuron pool [75], is highly coherent with the magnitude of isometric force fluctuations [76,77].

Importantly, the strength of common synaptic input to motor neurons has been demonstrated to increase during fatiguing contractions [17,78], indicating the need for greater neural drive to muscle, and providing an explanation for the neuromuscular fatigue-induced increase in the magnitude of force fluctuations. However, the exact mechanism(s) responsible for the increase in common synaptic input still remain(s) to be elucidated. Motor neurons receive synaptic input from three general sources: afferent feedback, neuromodulatory pathways from the brainstem, and descending cortical and reticulospinal pathways [79]. It is likely that neuromuscular fatigue affects each of these three sources, though the extent to which each of them contributes to the increase in common synaptic input, and the consequent loss of force control, remains to be determined, and is likely to be task-dependent. Once we know more about changes in the sources of synaptic input to motor neurons during neuromuscular fatigue, it may be possible to design interventions that attenuate such changes and serve to maintain force control, and, therefore, task performance for longer [79]. This, therefore, represents an important future line of research.

### 4.1. Afferent Feedback

Metabolite-mediated peripheral fatigue has been speculated to be a prerequisite for the increase in common synaptic input and loss of force control [18,22,25]. Contractions performed above the CT lead to the accumulation of metabolic by-products, e.g., inorganic phosphate, H^+^ and/or K^+^ [80]. Group IV muscle afferents are sensitive to such metabolites, and increase their discharge rates in response to increases in their levels, while group III muscle afferents are sensitive to muscle contraction and stretch [81]. Increases in group III and IV afferent feedback have been speculated to lead to motor neuron inhibition [82], which necessitates a compensatory increase in motor unit recruitment in order to maintain the demands of the task [83]. The accumulation of metabolic by-products also decreases the total area of motor unit twitch force [84], which is compensated for by an increase in motor unit discharge rate [83]. Such changes in motor unit recruitment and discharge rates likely reflect changes in the common synaptic input received by the motor unit pool as contractions progress and fatigue develops [17].

In contrast to group III and IV muscle afferents which increase their firing in response to neuromuscular fatigue, group Ia muscle afferents decrease their firing [85]. This suggests a progressive disfacilitation of the motor unit pool, which may contribute to the initial decrease in motor unit firing rates seen during fatiguing contractions. Moreover, it has been suggested that group III and IV muscle afferents are able to presynaptically inhibit Ia afferent input to motor neurons [86]. Further evidence for the role of Ia muscle afferent feedback comes from the observation that vibrotactile stimulation of muscle enhances excitatory input from Ia afferents to the motor unit pool [87], and decreases variability in both the cumulative motor unit spike train and force output [88]. It must be noted, though, that the firing of Ia muscle afferents differs between concentric, eccentric and isometric contractions [89] and, as such, their impact on common synaptic input is likely to vary, depending on contraction type (i.e., the isometric contractions typical of laboratory studies vs. the dynamic contractions typical of sporting and everyday actions).

### 4.2. Neuromodulatory Pathways

The intrinsic excitability of motor neurons is primarily controlled by neuromodulatory inputs (monoaminergic projections) that originate in the brainstem [90]. Animal and human studies have demonstrated that serotonin is released from the raphe-spinal pathway during intense motor activity [91]. This serotonergic projection has the potential to have a profound impact on motor neuron excitability, increasing the efficacy of synaptic inputs by three- to five-fold [92]. It has recently been demonstrated that ingestion of paroxetine, a selective serotonin reuptake inhibitor which increases the availability of serotonin, decreased muscle force CV (i.e., increased steadiness) during contractions in unfatigued muscle, and during a two-minute fatiguing contraction at 20% of MVC [93]. Given the coherence between muscle force CV and the cumulative motor unit spike train, differences in CV between conditions have been suggested to reflect differences in common synaptic input [79]. Enhanced serotonin availability may, therefore, have attenuated the increase in common synaptic input seen with neuromuscular fatigue. Such findings suggest that selective serotonin reuptake inhibitors could be used as ergogenic aids. One limitation of this study was the use of a fixed duration fatiguing contraction, rather than one performed until task failure. This may be relevant, because the authors previously demonstrated that enhanced serotonin availability amplifies perceptions of fatigue [91], an observation which may limit the potential of selective serotonin reuptake inhibitors as an ergogenic aid. It must be noted that serotonin is not the only neuromodulator capable of influencing muscle force control. Dopamine release is also increased with physical activity [94], and may influence muscle force control. In unfatigued muscle, antagonism of the D_2_ receptor increases muscle force CV during low- and moderate-intensity contractions [95].

### 4.3. Cortical and Reticulospinal Pathways

Transcranial magnetic stimulation (TMS) has frequently been used to assess supraspinal fatigue [96], identifying changes in corticospinal excitability and inhibitory processes. When recorded during rest following fatiguing exercise, a reduction in corticospinal excitability has commonly been shown [97,98]. The reduction in corticospinal excitability has also been accompanied with a lengthening in the corticospinal silent period (CSP), representing an increase in cortical and/or spinal inhibition [99]. Conversely, an increase in corticospinal excitability has been demonstrated in the preparation of precise force control tasks [100]. It is, therefore, logical to suggest that the reduced corticospinal excitability and increase in inhibition induced by fatiguing exercise may be one of the mechanisms responsible for fatigue-induced loss of muscle force control, although this has yet to be tested. Finally, with the important role that the reticulospinal tract has in balance [101], future research should consider the role that this pathway has on fatigue-induced loss of muscle force control.

## 5. Performance Implications of the Neuromuscular Fatigue-Induced Loss of Muscle Force Control

The neuromuscular fatigue-induced increase in the magnitude and decrease in the complexity of force fluctuations are reflective of decreased force steadiness and neuromuscular system adaptability, respectively. Such changes have been speculated to negatively impact motor control, movement mechanics and coordination [102], which could, in turn, increase the risk of failing motor tasks [22], increase risk of injury [103] and decrease the performance of skilled movements in sporting events [104] and functional tasks in everyday life [105]. Consequently, and given the ubiquity of fatigue in both daily life and sporting contexts [106], the neuromuscular fatigue-induced loss of force control has important implications for a wide range of populations. It must be noted, though, that no empirical research has directly investigated the relationship between the neuromuscular fatigue-induced loss of force control, and the performance indices mentioned above, or injury risk. Establishing whether the neuromuscular fatigue-induced loss of force control does indeed have an effect on subsequent task performance and injury risk, is a vital next step for research.

Research on unfatigued muscle has demonstrated that muscle force CV during low-intensity contractions of the plantarflexors [107], dorsiflexors and hip abductors [20] explains significant variance in static balance. In these studies, lower muscle force CV (i.e., greater steadiness) was associated with smaller centres of pressure displacements (i.e., less postural sway). Furthermore, lower knee extensor CV and greater SampEn have been associated with greater reach in the Y balance test, indicating greater dynamic balance [21]. Similar relationships have also been observed for wrist extensor CV and manual dexterity tasks [108], and for knee extensor CV and indices of locomotion [109]. Such results suggest, therefore, that the neuromuscular fatigue-induced loss of force control (i.e., greater CV, lower SampEn) should result in predictable decreases in the performances of such tasks [21]. However, as previously mentioned, no study has empirically tested whether the neuromuscular fatigue-induced loss of force control is a predictor for decreased balance, manual dexterity, or locomotion. Given that balance, manual dexterity and locomotion represent three fundamental motor skills [110], any effect of a loss of force control will have a significant impact on the performance of activities of daily living, occupational duties, and sporting performance.

It has been speculated that low muscle force complexity may be responsible, in part, for the inability to continue physical tasks (i.e., task failure) [18,22]. This is based on repeated observations that task failure (or exhaustion) during fatiguing contractions is associated with consistently low levels of complexity, regardless of experimental manipulation [14,18,22,47]. As low complexity reflects low adaptability in motor control, the consistently low values seen at task failure could be an adaptive mechanism to prevent further losses of complexity, which may lead to a greater risk of adverse outcomes, such as muscle damage or injury. This hypothesis provides a parallel with the “sensory tolerance hypothesis” [81], which proposes that metabolic perturbations that contribute to peripheral fatigue are detected by group III and IV afferents, which provide inhibitory feedback to the central nervous system; then, this reduces central motor drive, in order to restrict the development of peripheral fatigue beyond a certain limit [111]. Further support for a role of force control in task failure comes from the observations that baseline variability and complexity are correlated with endurance time, and increases the explanatory power of regression models for endurance time [70,71].

## 6. Conclusions

In this review, we have summarised research that demonstrated a neuromuscular fatigue-induced increase in the magnitude, and decrease in the complexity, of muscle force fluctuations (i.e., a loss of force control). Further research is needed to extend this loss of force control from typical laboratory fatigue tests (e.g., isometric contractions) to the contractions that are characteristic of everyday and sporting movements (e.g., dynamic concentric and eccentric contractions). The mechanistic basis for this loss of force control appears to be an increase in common synaptic input to motor neurons, though the exact contributions to this, of changes in afferent feedback, neuromodulatory pathways and cortical/reticulospinal pathways, remain to be determined. As muscle force control has important implications for activities of daily living, occupational duties and sporting performance, such fatigue-induced changes are likely to negatively effect performance in a wide range of populations. Further research is, however, needed to establish empirical relationships between this fatigue-induced loss of force control and functional/skilled movements.

## Data Availability

Not applicable.
